# Diagnostic accuracy of Pluslife MiniDock MTB on tongue swabs in sputum-scarce people with presumptive TB

**DOI:** 10.5588/ijtldopen.26.0182

**Published:** 2026-10-12

**Authors:** L. Rockman, S.M. Abdulgader, S. Minnies, D. Naidoo, A.M. Chiwaya, W. Nwamba, A. Okunola, G. Theron

**Affiliations:** ^1^DSI-NRF Centre of Excellence for Biomedical Tuberculosis Research, South African Medical Research Council Centre for Tuberculosis Research, Division of Molecular Biology and Human Genetics, Faculty of Medicine and Health Sciences, Stellenbosch University, Stellenbosch, South Africa;; ^2^Biomedical Research Institute (BMRI), Faculty of Medicine and Health Sciences, Stellenbosch University, Cape Town, South Africa.

**Keywords:** tuberculosis, MiniDock MTB, sputum-scarce, tongue swabs

Dear Editor,

A dependency on sputum limits access to scale-up of TB molecular testing,^1^ a public health priority. Promising tests, which can be done on tongue swabs (TSs) or sputum-dipped swabs, are WHO-endorsed.^2^ However, most data are from people who can expectorate sputum and are already testable using conventional tests. People unreachable by sputum tests, such as sputum-scarce people, stand to benefit the most from TSs and, in adults with symptoms, are thought to account for 13% of people.^3^ There are emerging data on TS test performance in sputum-scarce people. TS Xpert MTB/RIF Ultra (Ultra; Cepheid, USA) can double the ART-initiators diagnosed with TB compared with sputum testing alone.^4^ In hospitalised patients who require a bronchoscopy, TS testing can detect 80% of TB.^5^ These studies did not use a test designed for swabs like MiniDock MTB (Guangzhou Pluslife Biotech, China), the only near-point-of-care WHO-endorsed swab-based test. In a recent multi-country evaluation, MiniDock MTB on TSs had non-inferior diagnostic yield to sputum-based testing despite slightly diminished sensitivity on TSs versus sputum.^6^ This is because of TSs being more accessible than sputum. We are unaware of MiniDock MTB evaluations exclusively in sputum-scarce people.

We included sputum-scarce participants ≥12 years who had presumptive TB, defined per WHO^7^ and South African Department of Health guidelines^8^ (night sweats, cough [any duration if people living with HIV (PLHIV), ≥2 weeks if without HIV], weight loss, and fever; ≥1 symptom required if PLHIV, ≥2 if without HIV). People were classified as sputum-scarce if, after being asked to expectorate a sputum per Western Cape Provincial Department of Health guidance,^8^ no non-salivary sputum ≥1 mL was produced. Participants were consecutively recruited in the CAGE-TB (ClinicalTrials.gov NCT05317247) or SeroSelectTB (NCT04752592) studies in Cape Town, South Africa, from primary care prior to treatment initiation. We included everyone with a dry biobanked flocked swab (FLOQSwabs code 520C; Copan, Brescia, Italy) and a paired raw induced sputum collected on the same day and stored at −20°C. At enrolment, all sputum-scarce people had sputum induced. TSs were collected prior to sputum using the manufacturer-recommended procedure.^9^ Participants were classified as positive for TB using a microbiological reference standard (MRS) if, from a single Ultra and/or single MGIT960 on fresh induced sputum at enrolment (with positive growth speciated using Ultra), either was positive for *Mycobacterium tuberculosis* complex. Participants were classified as not having TB if at least one reference test was negative and neither Ultra nor culture was positive; participants with a missing culture or Ultra result were classified as not having TB if the available result was negative.

TSs from 86 people recruited between 4 July 2022 and 26 March 2025 were identified (70% [60/86] from CAGE-TB, remainder from SeroSelectTB). Paired induced sputum was available for 93% (80/86). After a median (interquartile range [IQR]) storage of 10 months (5–23), TSs were tested with MiniDock MTB with a minor instructions-for-use deviation as collection was prior to test availability. This change was necessary to accommodate stored swabs’ shorter breakpoint (30 vs. 80 mm for Pluslife-supplied swabs; https://doi.org/10.5281/zenodo.22108992). The rest of the procedure was per instructions-for-use,^10^ as was Pluslife testing of induced sputum with the addition of homogenisation using vortexing for 60 s prior to swab dipping. The sputum tested with Pluslife was separate from the induced sputum used for the MRS but was still collected the same day. STARD guidelines were followed (https://doi.org/10.5281/zenodo.22108992).^11^ Diagnostic accuracy metrics were calculated (https://doi.org/10.5281/zenodo.22108992). This work was approved by the Health Research Ethics Committee of Stellenbosch University (M20/06/017, M21/10/022), City of Cape Town (9517), and Western Cape Department of Health (202203_035). Written informed consent was obtained.

A total of 22% (19/86) had TB per the MRS and 20% (17/86) previous TB. The median (IQR) age was 35 (26–46), 63% (54/86) were female, and 31% (27/86) were PLHIV. TSs and induced sputum-dipped swabs had similar sensitivity (53% [95% confidence interval: 29, 76] vs. 53% [28, 77]; *P* = 0.985) and specificity (97% [90, 100] vs. 97% [89, 100]; *P* = 0.950) – see Figure and Table. Sensitivity and specificity for each specimen type did not differ by previous TB or HIV statuses; however, TS specificity was reduced in PLHIV compared with HIV uninfected people (91% [71, 99] vs. 100% [92, 100]; *P* = 0.040). The proportion of people with positive results on both TS and induced sputum-dipped swab by induced sputum Ultra semi-quantitation category is provided in Table S1 (https://doi.org/10.5281/zenodo.22108992).

**Figure.**
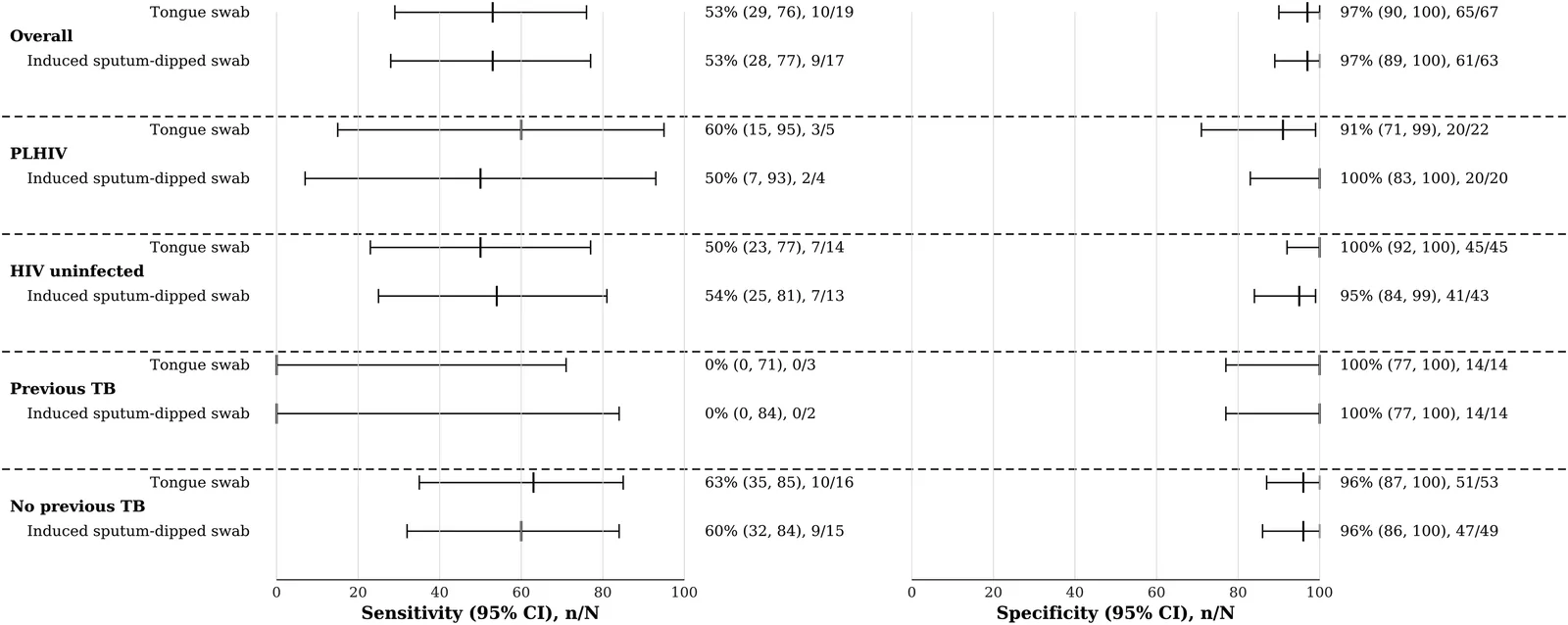
Forest plot of MiniDock MTB sensitivity and specificity on tongue and induced sputum-dipped swabs in sputum-scarce people, overall and stratified by HIV and previous TB status. Performance was similar between the two specimen types and across different groups; however, reduced specificity was observed for tongue swabs in people living with HIV (PLHIV) compared with HIV uninfected people. CI = confidence interval.

**Table.** Diagnostic accuracy of MiniDock MTB on TSs and induced sputum-dipped swabs compared with the MRS for the detection of TB stratified by HIV, and separately, previous TB status.

	Overall**^A^** (n = 86)		PLHIV (n = 27)		HIV uninfected (n = 59)		Previous TB (n = 17)		No previous TB (n = 69)	
	Sensitivity	Specificity	Sensitivity	Specificity	Sensitivity	Specificity	Sensitivity	Specificity	Sensitivity	Specificity
TS	53 (29, 76)	97 (90, 100)	60 (15, 95)	91 (71, 99)	50 (23, 77)	100 (92, 100)	0 (0, 71)	100 (77, 100)	63 (35, 85)	96 (87, 100)
n = 86/86	10/19	65/67	3/5	20/22	7/14	45/45	0/3	14/14	10/16	51/53
			*P* = 0.701*	*P* = 0.040*			*P* = 0.087*	*P* = 0.461*		
Induced sputum-dipped swab	53 (28, 77)	97 (89, 100)	50 (7, 93)	100 (83, 100)	54 (25, 81)	95 (84, 99)	0 (0, 84)	100 (77, 100)	60 (32, 84)	96 (86, 100)
	9/17	61/63	2/4	20/20	7/13	41/43	0/2	14/14	9/15	47/49
n = 80/86	*P* = 0.985^€^	*P* = 0.950^€^	*P* = 0.640^€^	*P* = 0.167^€^	*P* = 0.842^€^	*P* = 0.143^€^	*P* > 0.999^€^	*P* > 0.999^€^	*P* = 0.886^€^	*P* = 0.936^€^
			*P* = 0.720*	*P* = 0.327*			*P* = 0.206*	*P* = 0.442*		

Data are %, (95% CI) and n/N. Bold *P* values highlight significance: *P* < 0.05. Within row *P* values: *compared across HIV and previous TB status. Within column *P* values: ^€^TS vs. induced sputum swab.

Two sputum Ultra error results were reported and upon repeat Ultra testing done, resolved to one being MTB detected and the other, MTB not detected.

CI = confidence interval; MRS = microbiological reference standard (a single Ultra and/or single MGIT960, performed on fresh induced sputum at enrolment with positive growth speciated with Ultra, either was positive for *M. tuberculosis* complex); TS = tongue swab.

A Six people had no MiniDock MTB induced sputum swab result.

Our key finding is that TSs tested using MiniDock MTB in people who, even in a well-resourced research environment, could not expectorate sputum would approximately halve the amount of sputum-scarce TB missed. In other words, our TS sensitivity estimates here (which are lower than those reported previously,^6^ which included primarily sputum-productive people with advanced disease) are most appropriately compared with the typical programmatic scenario where sputum-scarce people are unable to access a rapid molecular test (53% vs. 0%). Our findings also suggest that MiniDock MTB sensitivities in TSs and induced sputum were similar, which makes sputum induction harder to justify in programmatic care in the TS era, unless a more sensitive test (e.g., culture) is applied. Notably, MiniDock MTB sensitivity in induced sputum was lower than that reported previously for expectorated sputum,^6^ potentially reflecting dilution due to nebulisation. We also identified diminished specificity in PLHIV compared with HIV uninfected people after stratification by previous TB (a known cause of sputum nucleic acid amplification test [NAAT] false-positivity);^12^ however, given the small sample size and number of PLHIV and the resulting wide confidence interval, this observation should be interpreted cautiously. This also applies to the sub-analysis by previous TB status. Long-term follow-up of such ‘false-positive’ people is needed.

Our study has strengths and limitations. To rapidly generate data submittable to the WHO in 2025, we retrospectively tested specimens and, for TSs, this necessitated a minor instructions-for-use deviation in consultation with the manufacturer. A single culture result was included in the reference standard and, although sputum Ultra was also included, a two-culture reference standard would be optimal. With specimens used for Pluslife (sputum, TSs) stored at −20°C, freeze–thaw cycles may degrade nucleic acids and reduce sensitivity or, alternatively, aid in cell lysis and release of material from the swab. Additionally, MiniDock MTB has not been validated on induced sputum, and the performance on this specimen type may differ from that on expectorated sputum. Given these limitations, prospective data from a larger cohort of sputum-scarce people are needed.

In conclusion, our data suggest that MiniDock MTB on TSs is a viable alternative to sputum induction, which is seldom available in programmatic conditions, and this can at least halve the amount of sputum-scarce TB missed in people with presumptive TB.
